# Supplementary material for: Soil moisture–atmosphere feedback dominates land carbon uptake variability
Source: Nature. 2021 Mar 31;592(7852):65–9. doi: 10.1038/s41586-021-03325-5 (PMC8012209; doi:10.1038/s41586-021-03325-5)
Supplement: Supplementary file 1 — This file contains Supplementary Methods, Supplementary Tables 1–4 and Supplementary Figures 1–16. [file 41586_2021_3325_MOESM1_ESM.pdf]

nature portfolio

https://doi.org/10.1038/s41586-021-03325-5

Supplementary information

Soil moisture–atmosphere feedback dominates land carbon uptake variability

In the format provided by the authors and unedited

Supplementary Materials for

Soil moisture–atmosphere feedback dominates land carbon uptake variability

Vincent Humphrey*, Alexis Berg, Philippe Ciais, Pierre Gentine, Martin Jung, Markus Reichstein, Sonia I. Seneviratne, Christian Frankenberg

correspondence to: vincent.humphrey [-at-] caltech.edu (vincent.humphrey [-at-] bluewin.ch)

This PDF file includes:

Supplementary Methods 1-2
Supplementary Tables 1-4
Supplementary Figures 1-16

Supplementary Methods 1: Analysis of changes in the components of NBP variance

In Figure 1, pairs of simulations (CTL and ExpA) are used to investigate changes in the variance of NBP using its decomposition into carbon uptake and release (NBP = GPP-ReD). As the correlation between GPP and ReD is generally different from zero, the variance (σ^2) of NBP corresponds the sum of the variance-covariance matrix of these two components:

$$\sigma^2(\mathrm{NBP}) = \sigma^2(GPP) + \sigma^2(ReD) + 2\sigma(GPP, ReD) \qquad \text{Eq. S1}$$

When evaluating the impact of changes in individual components on the NBP variance, two types of changes should be distinguished. On the one hand, there can be a change in the variance of an individual component, which will automatically impact the covariance between this component and all the other components. On the other hand, and independently of changes in the variance of a component, a change in the correlation between two components can impact the covariance between these components. These two different effects are identified as follows.

First, changes in the variance of each individual component are assessed, for instance:

$$\Delta\sigma^2(\mathrm{GPP}) = \sigma^2(\mathrm{GPP_{ExpA}}) - \sigma^2(\mathrm{GPP_{CTL}}) \qquad \text{Eq. S2}$$

$$\Delta\sigma^2(\mathrm{ReD}) = \sigma^2(\mathrm{ReD_{ExpA}}) - \sigma^2(\mathrm{ReD_{CTL}}) \qquad \text{Eq. S3}$$

Then, we calculate the covariance which would be expected in experiment ExpA if the correlation (r) between the components was the same in ExpA as it is in the CTL experiment:

$$\sigma_{\mathrm{expected}}(\mathrm{GPP_{ExpA}}, \mathrm{ReD_{ExpA}}) = \mathrm{r}(\mathrm{GPP_{CTL}}, \mathrm{ReD_{CTL}}) \cdot \sqrt{\sigma^2(\mathrm{GPP_{ExpA}})} \cdot \sqrt{\sigma^2(\mathrm{ReD_{ExpA}})}$$

Eq. S4

The impact of any change in the correlation between components on the covariance is calculated as the difference between the actual covariance in ExpA and the covariance expected in ExpA.

$$\Delta\, 2\sigma(\mathrm{GPP}, \mathrm{ReD}) = 2\sigma(\mathrm{GPP_{ExpA}}, \mathrm{ReD_{ExpA}}) - 2\sigma_{\mathrm{expected}}(\mathrm{GPP_{ExpA}}, \mathrm{ReD_{ExpA}})$$

Eq. S5

Equations S2, S3 and S5 indicate how the contributions illustrated inside the bars of Figure 1c are estimated.

Supplementary Methods 2: Estimation of NBP$^{\text{T\&VPD LAC}}$ and NBP$^{\text{T\&VPD NonLAC}}$ variances

In Figure 3, we separate the temperature and VPD–driven NBP variance $\sigma^2\left(NBP_{CTL}^{T\&VPD}\right)$, into a LAC-dependent contribution $\sigma^2\left(NBP_{CTL}^{T\&VPD\ LAC}\right)$ corresponding to indirect (feedback) soil moisture effects, and a non LAC-dependent contribution $\sigma^2\left(NBP_{CTL}^{T\&VPD\ NonLAC}\right)$ at the local (grid-cell) scale. These two contributions (LAC and NonLAC) are estimated as follows.

In the control experiment (CTL), NBP$^{\text{T\&VPD}}$ includes both of these two contributions (i.e. the LAC and non-LAC dependent contributions). By definition, the overall NBP$^{\text{T\&VPD}}$ variance in the CTL experiment is the sum of the variance of each of the two contributions and the covariance between them:

$$\sigma^2\left(\mathrm{NBP}_{\mathrm{CTL}}^{\mathrm{T}\&VPD}\right) = \sigma^2\left(\mathrm{NBP}_{\mathrm{CTL}}^{\mathrm{T}\&VPD\ \mathrm{LAC}}\right) + \sigma^2\left(\mathrm{NBP}_{\mathrm{CTL}}^{\mathrm{T}\&VPD\ \mathrm{NonLAC}}\right) + 2\ \sigma\left(\mathrm{NBP}_{\mathrm{CTL}}^{\mathrm{T}\&VPD\ \mathrm{LAC}}, \mathrm{NBP}_{\mathrm{CTL}}^{\mathrm{T}\&VPD\ \mathrm{NonLAC}}\right)$$

Eq. S6

The left term $\sigma^2\left(NBP_{CTL}^{T\&VPD}\right)$ can be computed directly (Eq. 1-2 in the main Methods section). The term $\sigma^2\left(NBP_{CTL}^{T\&VPD\ NonLAC}\right)$ corresponds to the part of the variance in NBP$^{\text{T\&VPD}}$ which is independent of LAC. It can be estimated from the NBP$^{\text{T\&VPD}}$ that still persists in ExpA:

$$\sigma^2\left(NBP_{CTL}^{T\&VPD\ NonLAC}\right) \approx \sigma^2\left(NBP_{ExpA}^{T\&VPD}\right) \qquad \text{Eq. S7}$$

Note that $NBP_{ExpA}^{T\&VPD}$ is obtained by fitting Eq. 1 (main Methods section) to the ExpA simulation, thus potential changes in the sensitivities ($\beta_{s,m}^{T}$ or $\beta_{s,m}^{VPD}$) between CTL and ExpA are also accounted for.

Rearranging Eq. S6 with Eq. S7 and solving for $\sigma^2\left(\mathrm{NBP}_{\mathrm{CTL}}^{\mathrm{T\&VPD\ LAC}}\right)$ yields:

$$\sigma^2\left(\mathrm{NBP}_{\mathrm{CTL}}^{\mathrm{T\&VPD\ LAC}}\right) = \sigma^2\left(\mathrm{NBP}_{\mathrm{CTL}}^{\mathrm{T\&VPD}}\right) - \sigma^2\left(\mathrm{NBP}_{\mathrm{ExpA}}^{\mathrm{T\&VPD}}\right) - 2\ \sigma\left(\mathrm{NBP}_{\mathrm{CTL}}^{\mathrm{T\&VPD\ LAC}}, \mathrm{NBP}_{\mathrm{CTL}}^{\mathrm{T\&VPD\ NonLAC}}\right)$$

Eq. S8

The remaining unknown in Eq. S8 is the covariance term $\sigma\left(NBP_{CTL}^{T\&VPD\ LAC}, NBP_{CTL}^{T\&VPD\ NonLAC}\right)$. With Eq. S7, this term can be written as:

$$\sigma\left(\mathrm{NBP}_{\mathrm{CTL}}^{\mathrm{T}\&VPD\ \mathrm{LAC}}, \mathrm{NBP}_{\mathrm{CTL}}^{\mathrm{T}\&VPD\ \mathrm{NonLAC}}\right) = \mathrm{r}\left(\mathrm{NBP}_{\mathrm{CTL}}^{\mathrm{T}\&VPD\ \mathrm{LAC}}, \mathrm{NBP}_{\mathrm{CTL}}^{\mathrm{T}\&VPD\ \mathrm{NonLAC}}\right) \cdot \sqrt{\sigma^2(\mathrm{NBP}_{\mathrm{CTL}}^{\mathrm{T}\&VPD\ \mathrm{LAC}})} \cdot \sqrt{\sigma^2(\mathrm{NBP}_{\mathrm{ExpA}}^{\mathrm{T}\&VPD})}$$

Eq. S9

Where the only remaining unknown (beyond $\sigma^2\left(\mathrm{NBP}_{\mathrm{CTL}}^{\mathrm{T\&VPD\ LAC}}\right)$) is the correlation between the LAC-dependent and the non LAC-dependent contributions. This correlation is constrained by the following inequality:

$$-1 \le \mathrm{r}\left(\mathrm{NBP}_{\mathrm{CTL}}^{\mathrm{T}\&VPD\ \mathrm{LAC}}, \mathrm{NBP}_{\mathrm{CTL}}^{\mathrm{T}\&VPD\ \mathrm{NonLAC}}\right) \le 1 \qquad \text{Eq. S10}$$

Thus, while it is not possible to estimate $\sigma\left(NBP_{CTL}^{T\&VPD\ LAC}, NBP_{CTL}^{T\&VPD\ NonLAC}\right)$ exactly, a range of possible values can be estimated, thus also giving a range of possible values for $\sigma^2\left(\mathrm{NBP}_{\mathrm{CTL}}^{\mathrm{T}\&VPD\ \mathrm{LAC}}\right)$.

The smallest (and most conservative) $\sigma^2\left(\mathrm{NBP}_{\mathrm{CTL}}^{\mathrm{T}\&VPD\ \mathrm{LAC}}\right)$ occurs if the correlation between LAC-dependent and non LAC-dependent effects is exactly 1. In this hypothetical case, the ecosystem sensitivity to a given change in temperature (or VPD) is assumed to be the same whether that change in temperature was itself dependent on LAC or not, and the effect of LAC on T and VPD anomalies is

assumed to be positive and linear. In Figure 3, we estimate $\sigma^2\left(\mathrm{NBP}_{\mathrm{CTL}}^{\mathrm{T}\&VPD\ \mathrm{LAC}}\right)$ with this conservative assumption that $\mathrm{r}\left(\mathrm{NBP}_{\mathrm{CTL}}^{\mathrm{T}\&VPD\ \mathrm{LAC}}, \mathrm{NBP}_{\mathrm{CTL}}^{\mathrm{T}\&VPD\ \mathrm{NonLAC}}\right)$ is equal to 1. Assuming a lower correlation would cause $\sigma^2\left(\mathrm{NBP}_{\mathrm{CTL}}^{\mathrm{T}\&VPD\ \mathrm{LAC}}\right)$ to become even larger relative to $\sigma^2\left(\mathrm{NBP}_{\mathrm{CTL}}^{\mathrm{T}\&VPD\ \mathrm{NonLAC}}\right)$, however, the impact on the overall results is limited (see Supplementary Fig. 16, which reproduces Figure 3b and Extended Data Fig. 7 but assumes a correlation of 0 between LAC-dependent and non LAC-dependent effects).

Supplementary Table 1. *Change in global mean NBP inter-annual variability between the CTL and ExpA experiments and attribution to changes in GPP and ReD variability. The two leftmost columns have units of PgC yr^{-1}. Values are computed from annual means (n=46 years).*

	$Var(NBP_{CTL})$	*$Var(NBP_{ExpA})$*	*Reduction*	*Contr. of ΔGPP Var*	*Contr. of ΔReD Var*	*Contr. of Δr(GPP,ReD)*
CCSM4	0.75	0.08	89.8%	59.4%	30.4%	10.2%
ECHAM6	2.42	0.17	92.9%	74.5%	18.2%	7.3%
GFDL	7.63	0.86	88.7%	83.0%	16.3%	0.7%
IPSL	2.09	0.14	93.4%	N/A	N/A	N/A
Mean	3.22	0.31	91.2% (2.3%)	72.3% (12%)	21.6% (7.7%)	6.0% (4.8%)

Supplementary Table 2. *Relative contribution to global mean inter-annual NBP^{*}_{CTL} variance, by meteorological driver. Values are computed from annual means (n=46 years).*

	$Var(NBP^{SM})$	*$Var(NBP^{T\&VPD\ LAC})$*	*$Var(NBP^{T\&VPD\ NonLAC})$*	*$Var(NBP^{R})$*
CCSM4	34.4%	40.0%	9.8%	17.8%
ECHAM6	17.8%	60.5%	8.3%	13.4%
GFDL	1.4%	82.1%	9.1%	7.4%
IPSL	24.9%	58.8%	9.9%	6.4%
Mean	19.6%	59.9%	9.3%	11.2%
*(*SD*)*	(13.9%)	(18.0%)	(0.7%)	(5.3)

Supplementary Table 3. *Net effect of suppressing direct and indirect SM effects on inter-annual NBP^{*}_{CTL} variance (taking into account the covariance between the components). This agrees with the raw model outputs (third column in Supplementary Table 1), even though the sensitivity analysis can slightly under- or overestimate the magnitude of the reduction. Values are computed from annual means (n=46 years).*

	$Var(NBP^{*}_{CTL})$	*$Var(NBP^{T\&VPD\ NonLAC}+NBP^{R})$*	*Reduction*
CCSM4	0.30	0.03	88.7%
ECHAM6	2.20	0.14	93.6%
GFDL	12.22	0.92	92.5%
IPSL	2.69	0.11	95.8%

Supplementary Table 4. *Data availability of carbon flux variables within GLACE-CMIP5. If some variables that were not saved can be calculated from other variables, this is indicated in parentheses. NBP, net biome production; GPP, gross primary production; NPP, net primary production; RA, autotrophic respiration; RH, heterotrophic respiration; Re, autotrophic and heterotrophic respiration; D, disturbances (fire and land use change if applicable); NEE, net ecosystem exchange; ReD, sum of Re and D.*

		CCSM4	*ECHAM6*	*GFDL*	*IPSL*
CTL	NBP	yes	yes	yes	yes
	GPP	yes	yes	yes	no
	NPP	yes	yes	no	no
	RA	yes	no (GPP-NPP)	yes	yes
	RH	yes	yes	no	yes
	Re	no (RA+RH)	no (RA+RH)	no	no
	D	no (NEE-NBP)	no (NEE-NBP)	no	no
	NEE	no (GPP-Re)	no (GPP-Re)	no	no
	ReD	no (GPP-NBP)	no (GPP-NBP)	no (GPP-NBP)	no
ExpA	NBP	yes	yes	yes	yes
	GPP	yes	yes	yes	no
	NPP	no (GPP-RA)	yes	no	no
	RA	yes	no (GPP-NPP)	yes	no
	RH	yes	yes	no	no
	Re	no (RA+RH)	no (RA+RH)	no	no
	D	no (NEE-NBP)	no (NEE-NBP)	no	no (NEE-NBP)
	NEE	no (GPP-Re)	no (GPP-Re)	no	yes
	ReD	no (GPP-NBP)	no (GPP-NBP)	no (GPP-NBP)	no

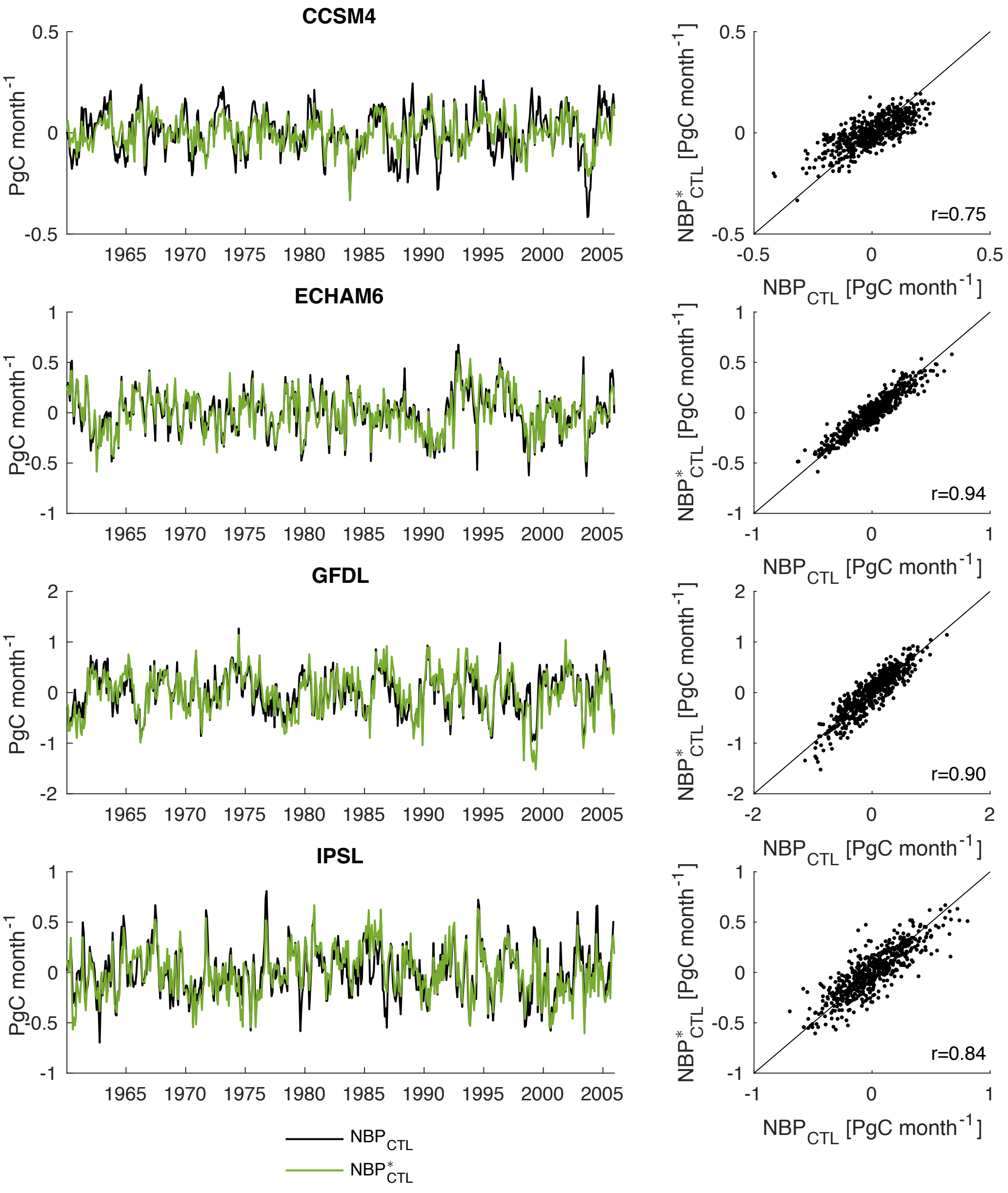

Supplementary Figure 1. Evaluation of the statistical sensitivity analysis (see Methods) for the control experiment (CTL). Each row depicts time series of the original global mean NBP (NBP_{CTL}) and the result of the global mean NBP (NBP^*_{CTL}) from the local month-wise regression (Eq. 1) against the main climatic drivers, at monthly resolution. The scatter plots depict the agreement between both time series (n=552 months).

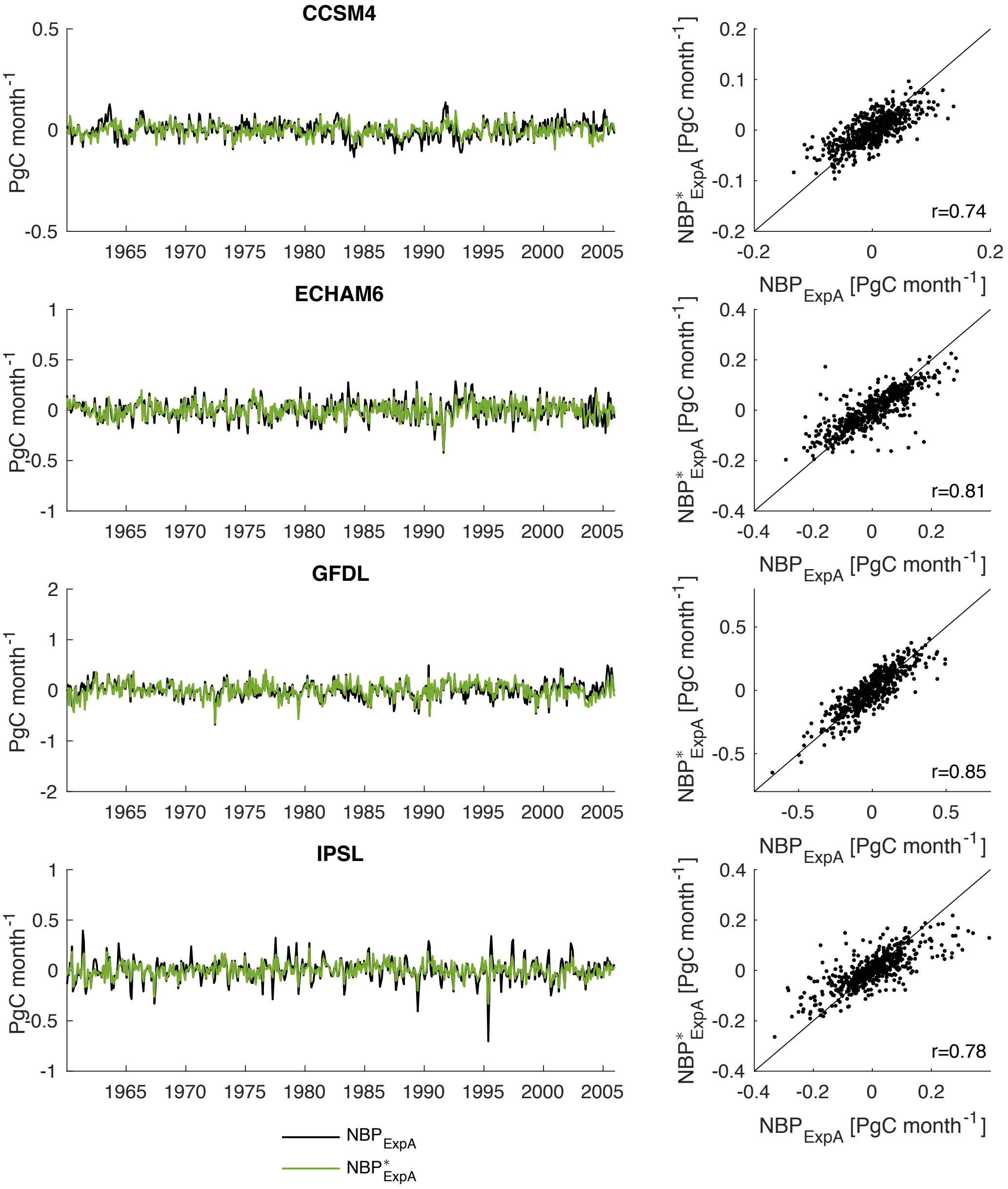

Supplementary Figure 2. Evaluation of the statistical sensitivity analysis (see Methods) for the experiment with no soil moisture inter-annual variability (ExpA). Each row depicts time series of the original global mean NBP (NBP_{ExpA}) and the result of the global mean NBP (NBP^*_{ExpA}) from the local month-wise regression (Eq. 1) against the main climatic drivers, at monthly resolution. The scatter plots depict the agreement between both time series (n=552 months).

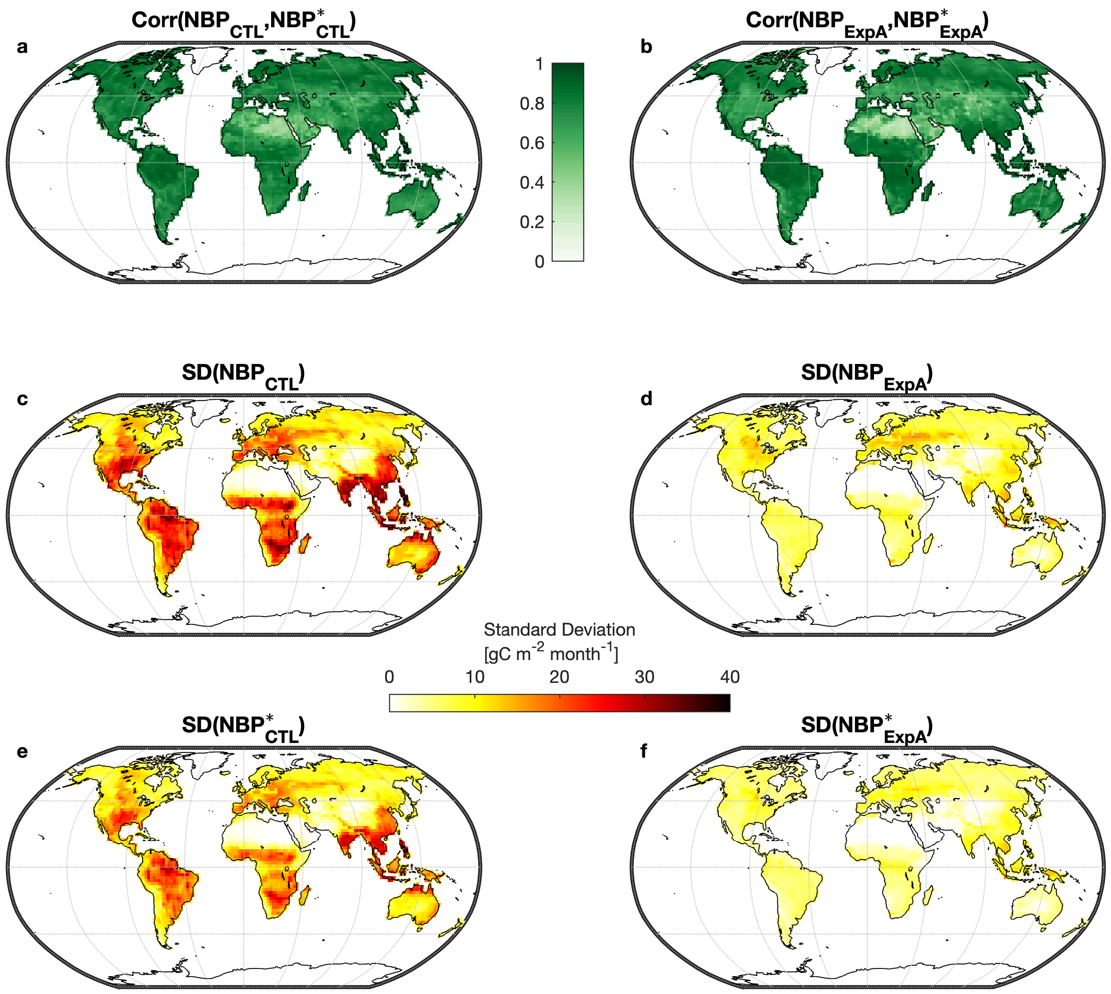

Supplementary Figure 3. Evaluation of the sensitivity analysis approach (see Methods) for both CTL and ExpA at the regional scale. a-b) Correlation between NBP and NBP for the CTL and ExpA simulations. c,e) Standard deviation (SD) of NBP and NBP* for the CTL simulation. d,f) Standard deviation of NBP and NBP* for the ExpA simulation. The median of the models is shown.*

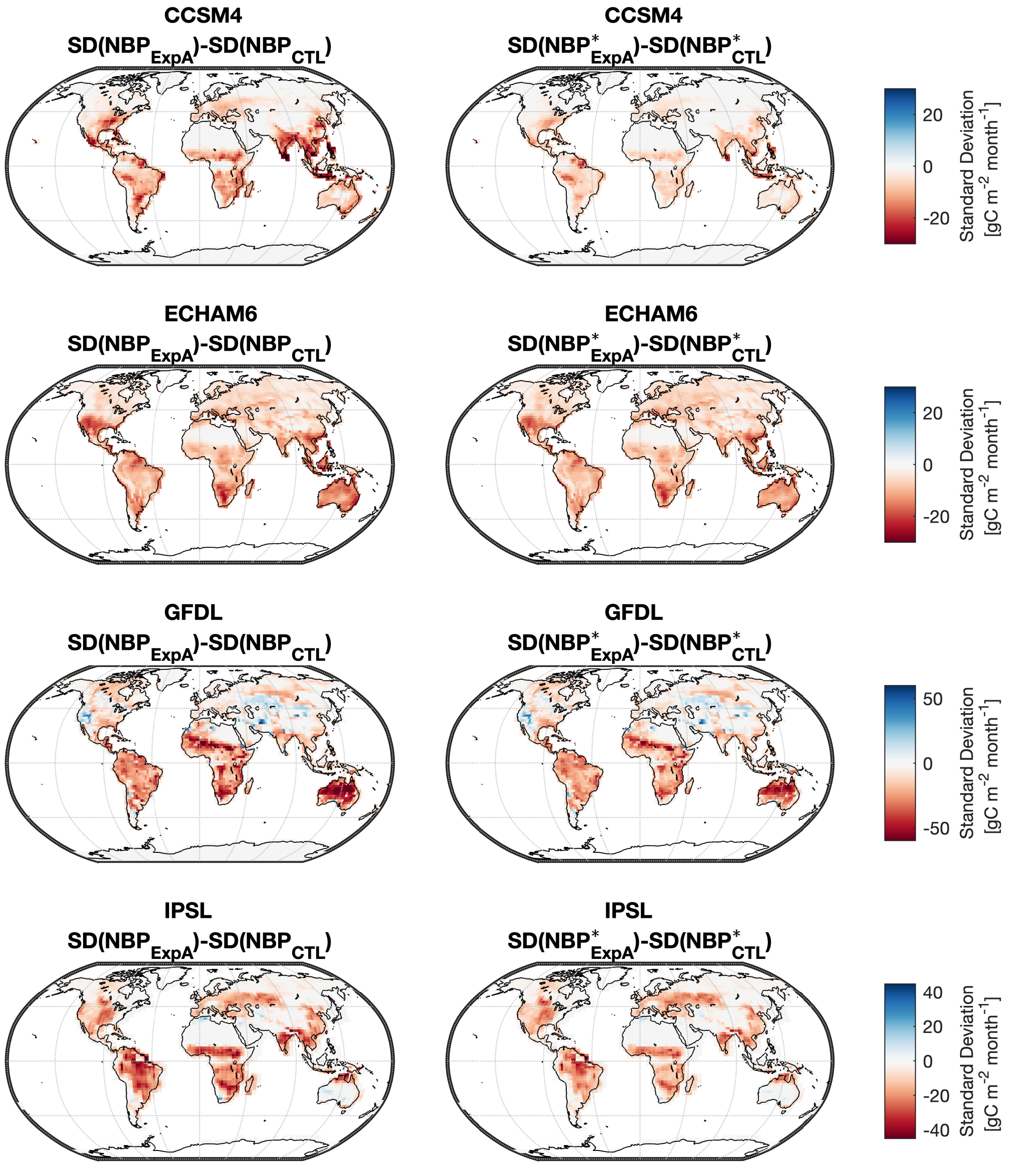

Supplementary Figure 4. Evaluation of the ability of the statistical sensitivity analysis (see Methods) to reproduce the difference in standard deviation (SD) between the CTL and the ExpA simulations. Left: Difference based on the raw climate model simulations. Right: Difference estimated from the sensitivity analysis (NBP). Each row corresponds to one of the four climate models employed in the study.*

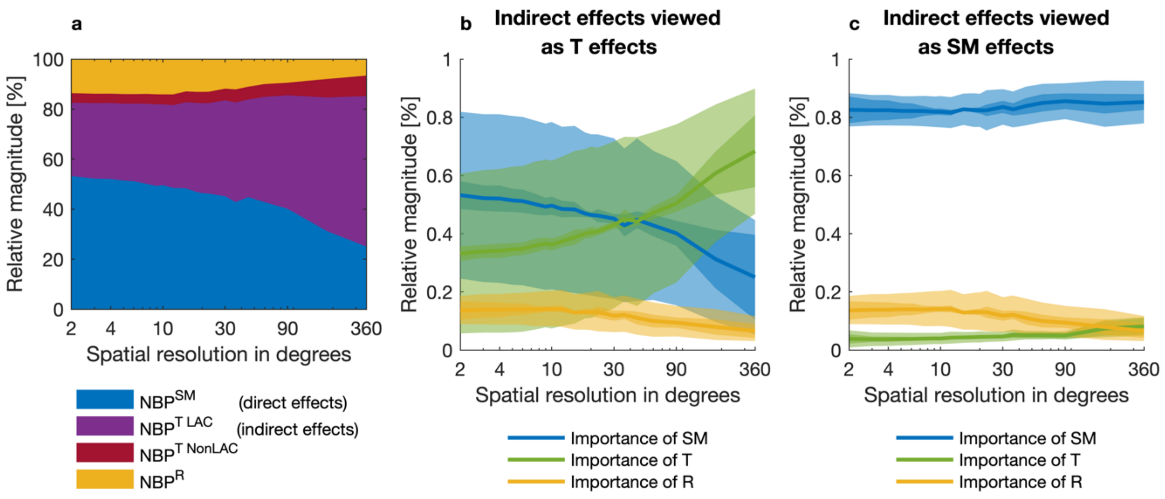

Supplementary Figure 5. ***a)*** *Same Extended Data Fig. 7 but using another formulation for the sensitivity analysis ($NBP = NBP^{SM}+NBP^{T}+NBP^{R}$), which neglects VPD. This formulation is directly comparable to Jung et al.*[2] *and* ***(b)*** *reproduces their findings. For a discussion of the different formulations, refer to the Methods section "Sensitivity analysis".*

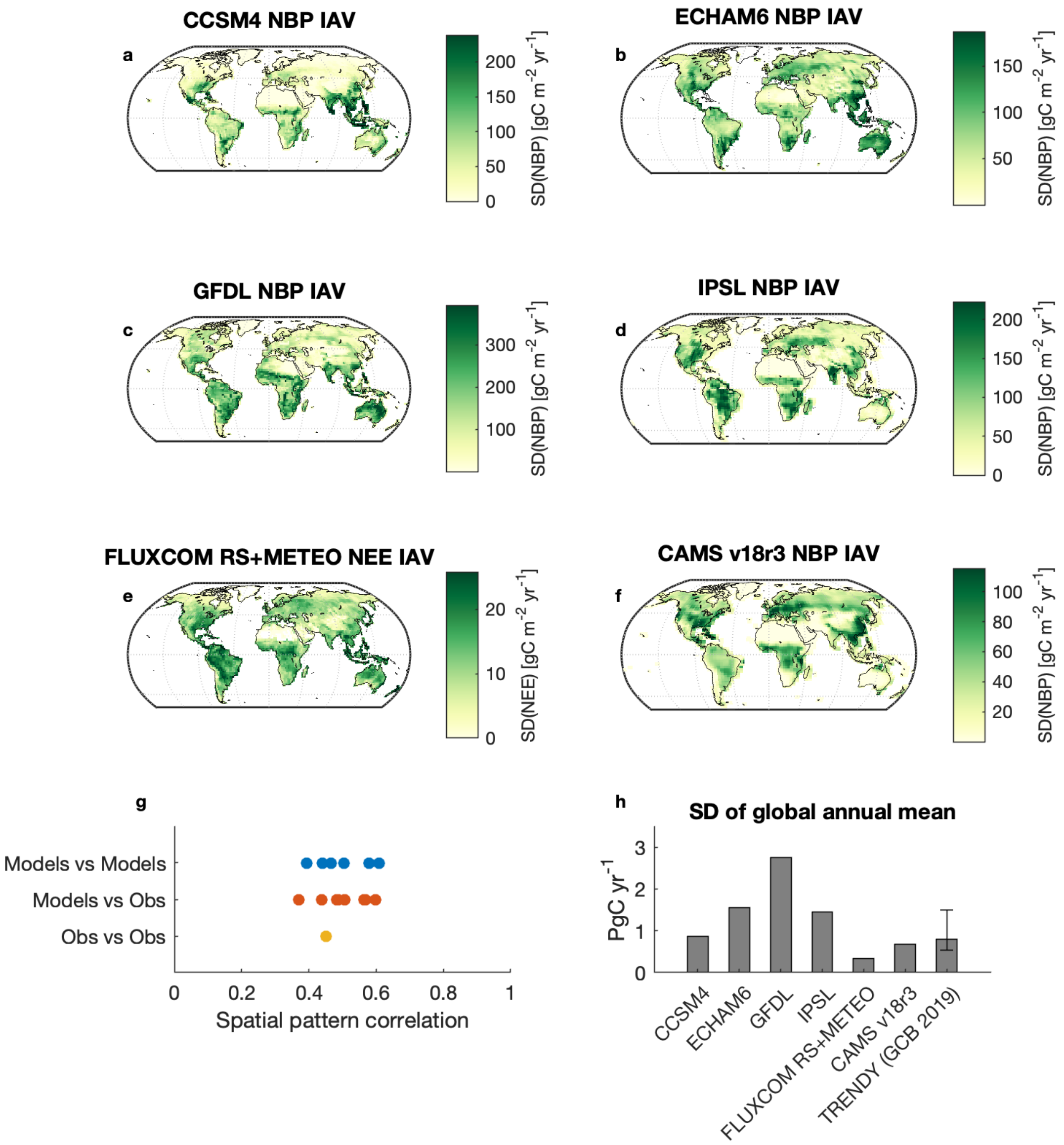

Supplementary Figure 6. ***(a-d)*** *NBP IAV (de-trended) as simulated by models in the control experiment.* ***(e)*** *NBP IAV (de-trended) as estimated from the FLUXCOM RS+METEO (GSWP3 forcing) dataset, which is a machine-learning-based upscaling of flux tower observations (period 1980-2010).* ***(f)*** *Biospheric fluxes estimated from the CAMS v18r3 atmospheric CO_2 inversion (period 2000-2018).* ***(g)*** *Spatial pattern correlation between the different data sources shown in (a-f).* ***(h)*** *global NBP IAV (de-trended) as estimated by the different products, as well as by the TRENDY dynamic global vegetation models (DGVMs) used in the Global Carbon Budget 2019. The error bar for the TRENDY models indicate the minimum and maximum value among the different DGVMS.*

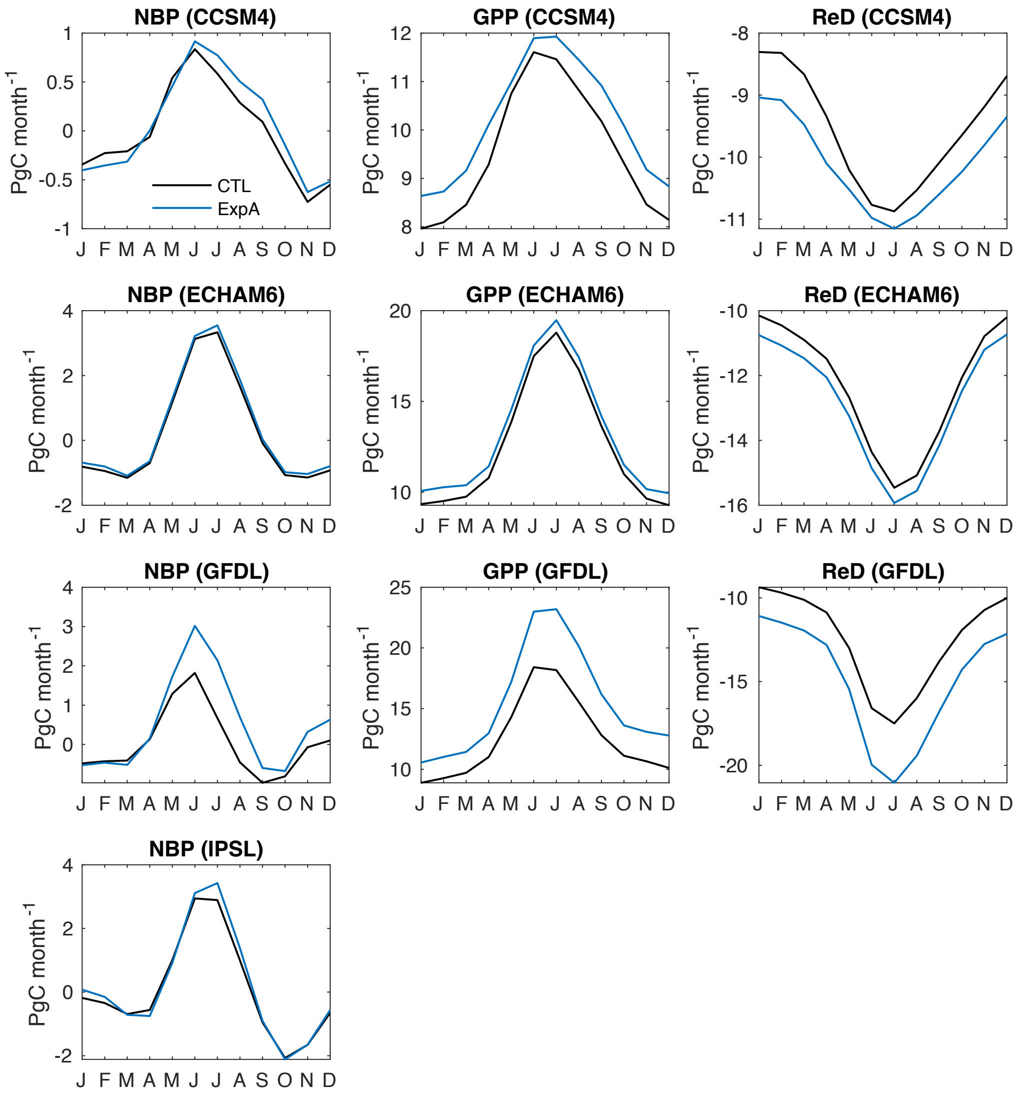

Supplementary Figure 7. Seasonal cycles of global mean NBP, GPP and ReD simulated by the four climate models used in this study. The GPP and ReD outputs are not available for the IPSL model (see Supplementary Table 4). Positive values indicate a flux from the atmosphere to the land (uptake).

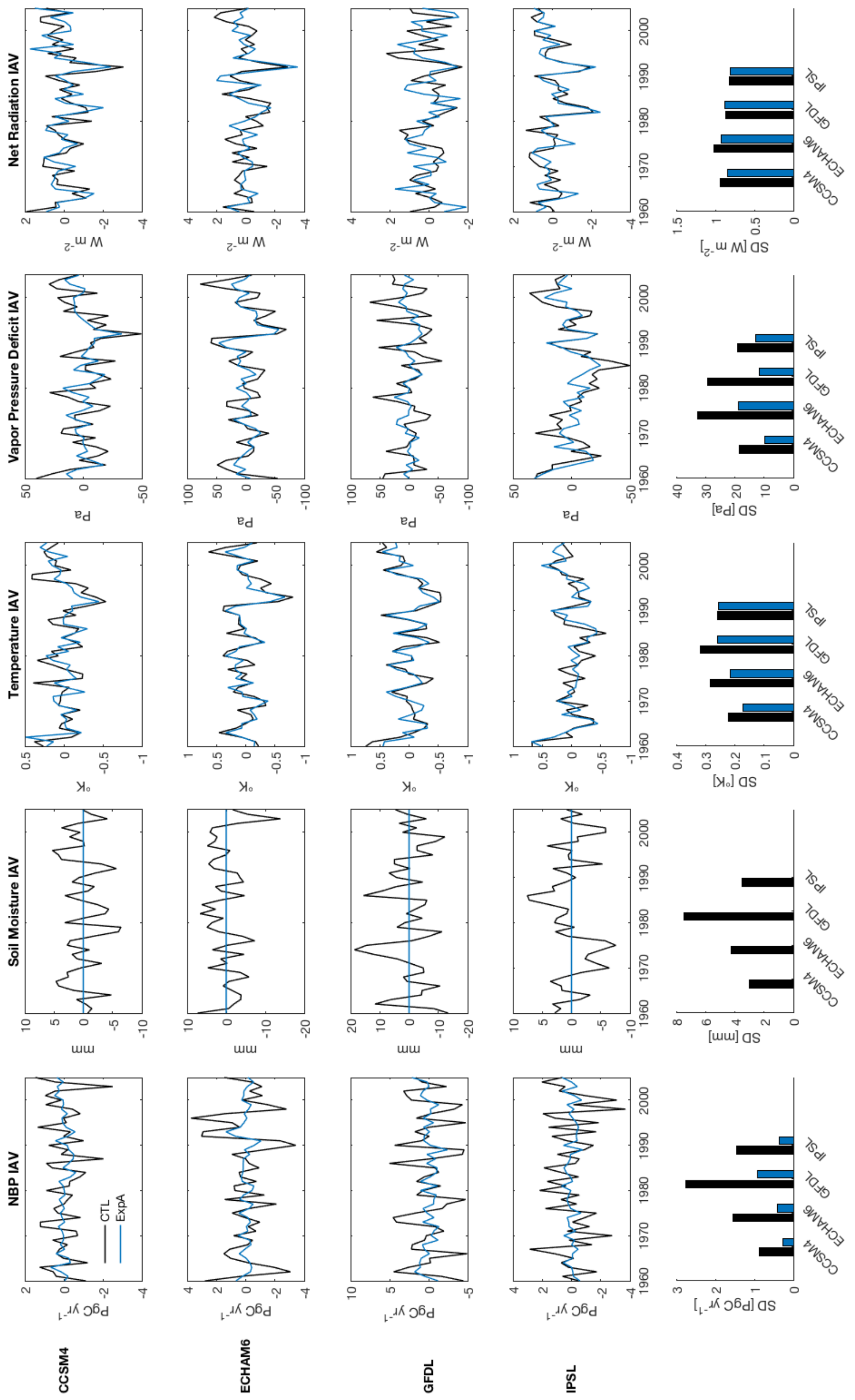

Supplementary Figure 8. Global mean IAV of NBP, SM, T, VPD and radiation, as simulated by the four climate models used in this study. The CTL time series (black) is the reference run, the ExpA time series (blue) is the simulation with prescribed seasonal soil moisture (suppressed soil moisture variability). The bottom row shows the standard deviation of these time series.

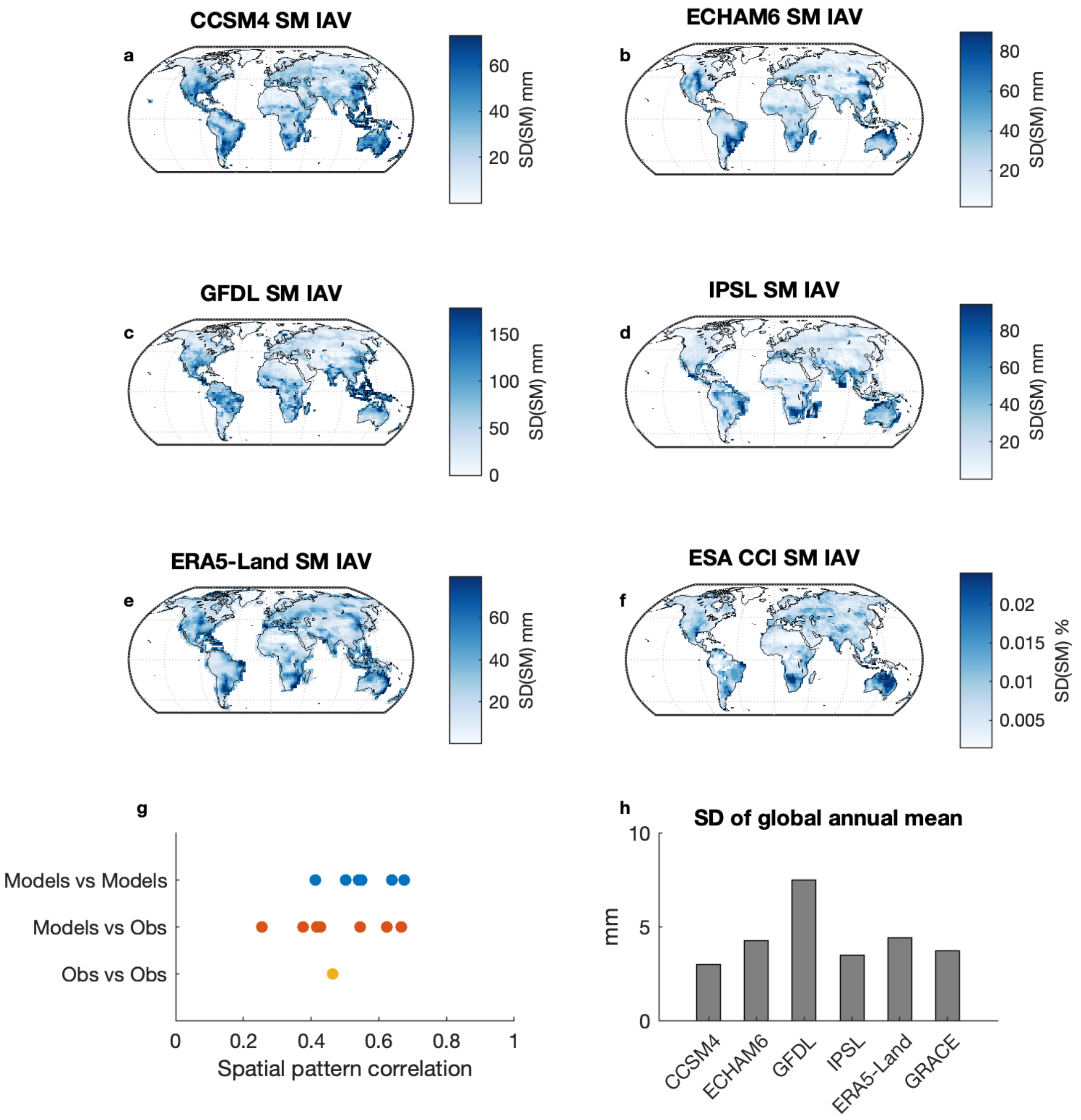

Supplementary Figure 9. ***(a-d)*** *Soil moisture IAV as simulated by models in the control experiment.* ***(e)*** *Soil moisture IAV as estimated from ERA5-Land, which is a land surface model forced with bias-corrected historical weather data.* ***(f)*** *Soil moisture IAV as estimated by the ESA CCI v4.5 combined satellite product, which measures shallow soil moisture (top 5 to 10 cm).* ***(g)*** *Spatial pattern correlation between the different data sources shown in (a-f).* ***(h)*** *global soil moisture IAV (and terrestrial water storage, from the GRACE JPL mascons satellite data), as estimated by different datasets.*

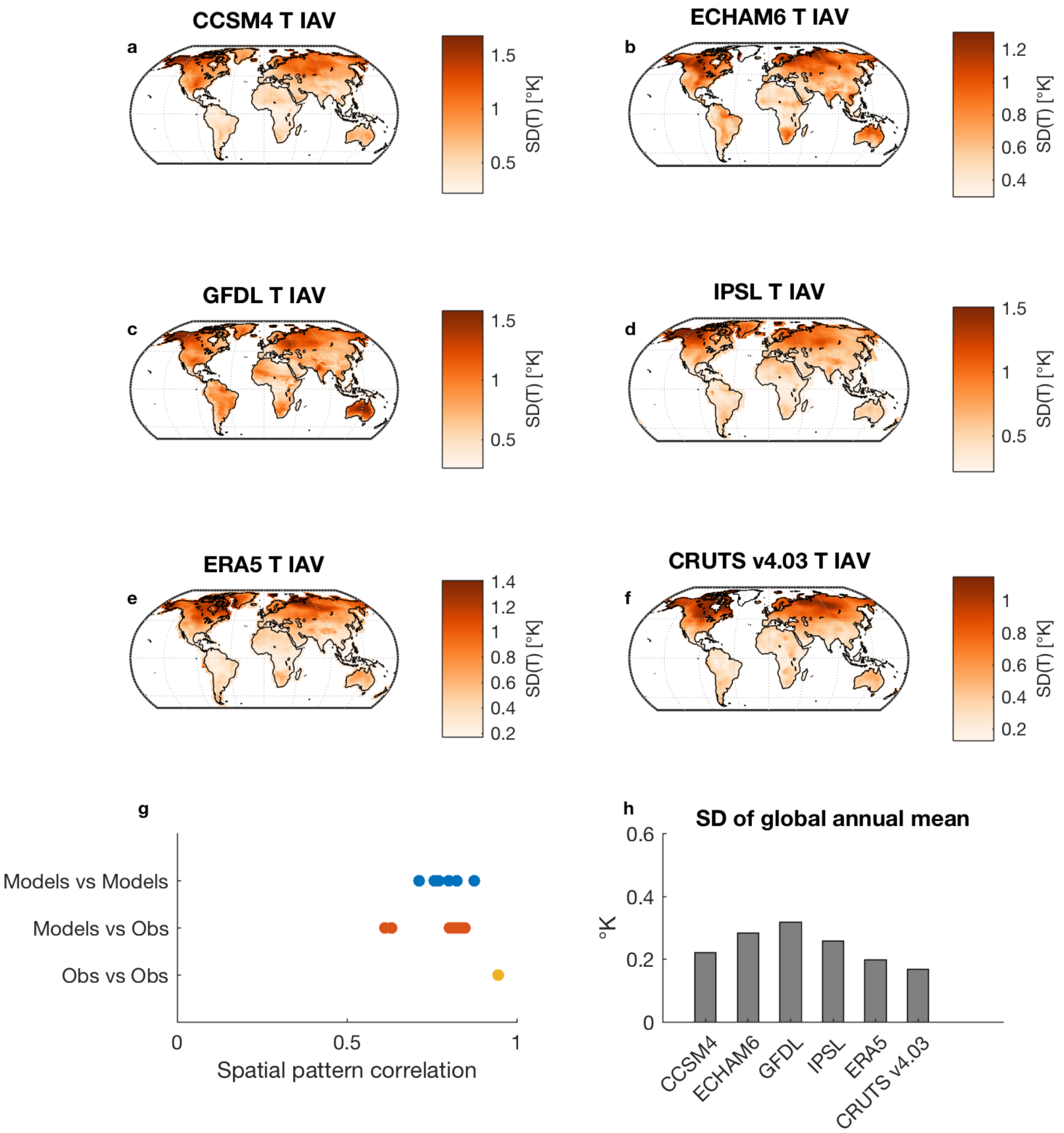

Supplementary Figure 10. ***(a-d)*** *Temperature IAV as simulated by models in the control experiment.* ***(e)*** *Temperature IAV as estimated from ERA5, which is an atmospheric reanalysis.* ***(f)*** *Temperature IAV as estimated from the CRU dataset, which is based on in-situ weather station measurements.* ***(g)*** *Spatial pattern correlation between the different data sources shown in (a-f).* ***(h)*** *global temperature standard deviation as estimated by the different datasets (de-trended data).*

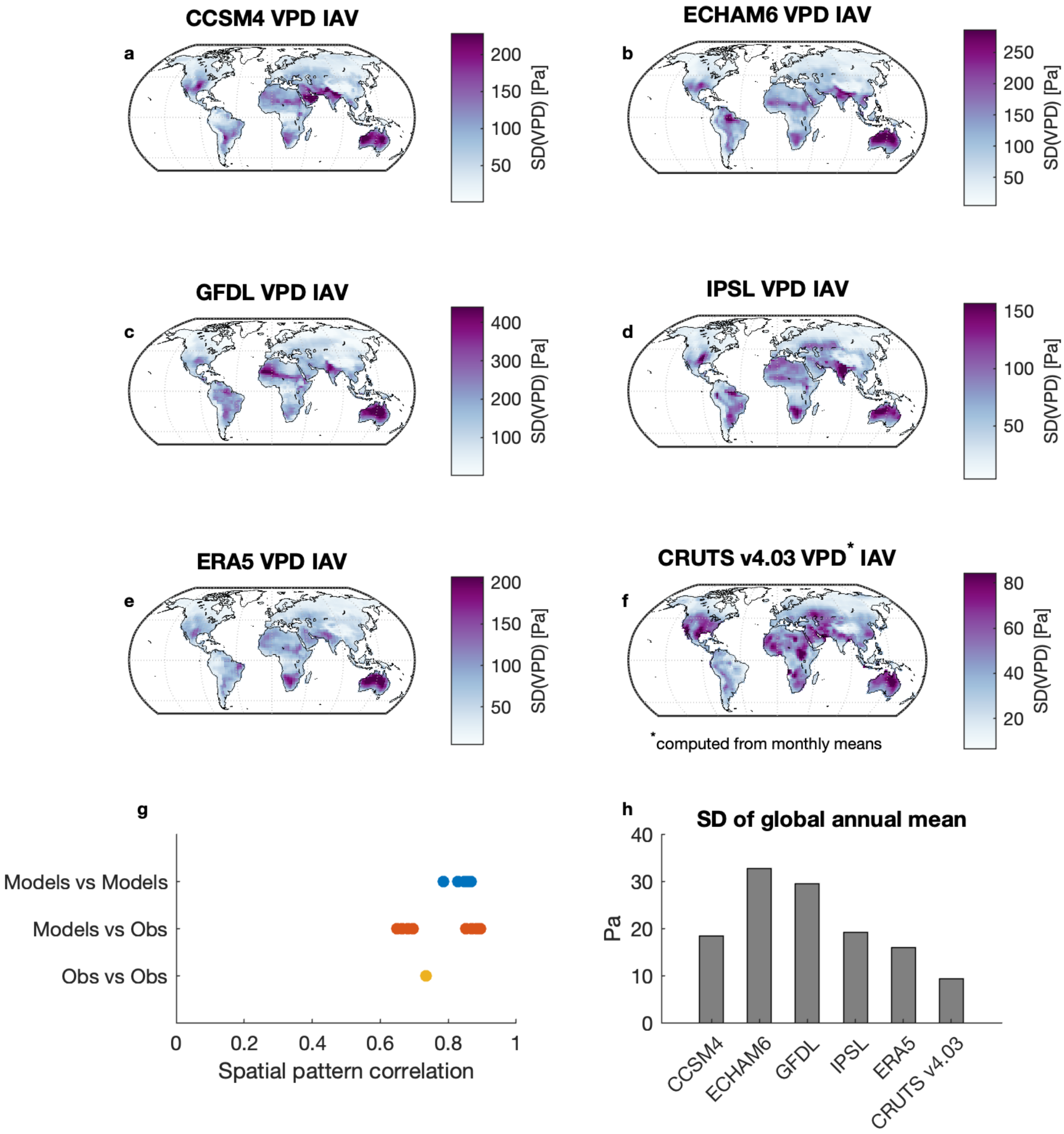

Supplementary Figure 11. ***(a-d)*** *VPD IAV as simulated by models in the control experiment.* ***(e)*** *VPD IAV as estimated from ERA5, which is an atmospheric reanalysis.* ***(f)*** *VPD IAV as estimated from the CRU dataset, which is based on in-situ weather station measurements. As CRU VPD is computed from monthly means of temperature and humidity (instead of daily means), it should be expected to exhibit a lower amount of variability overall.* ***(g)*** *Spatial pattern correlation between the different data sources shown in (a-f).* ***(h)*** *global SM (or TWS) IAV as estimated by the different datasets.*

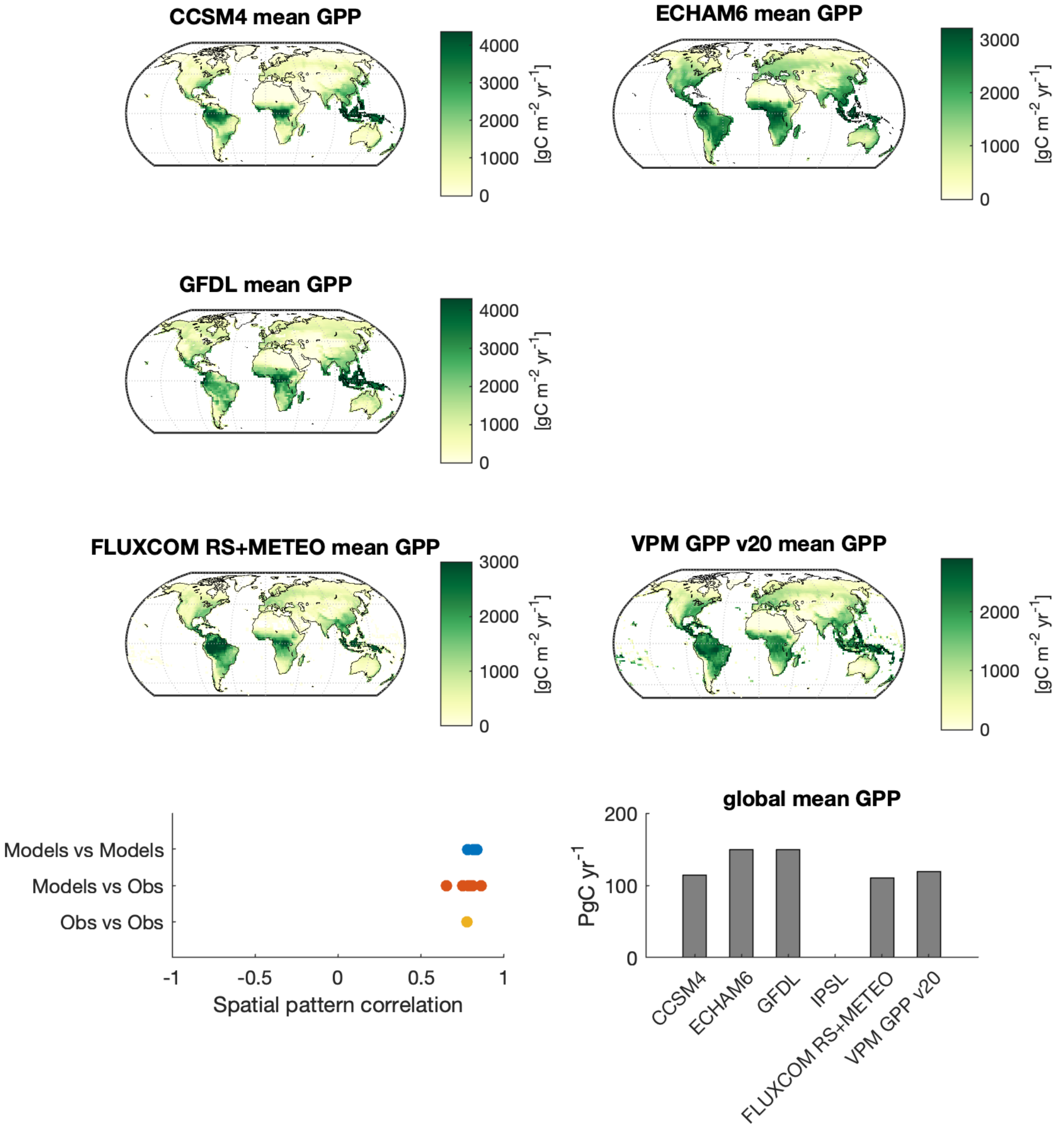

Supplementary Figure 12. ***(a-d)*** *Annual GPP as simulated by models in the control experiment (not available for IPSL).* ***(e)*** *Annual GPP as estimated from FLUXCOM RS+METEO (GSWP3 forcing) dataset, which is a machine-learning-based upscaling of flux tower observations.* ***(f)*** *Annual GPP as estimated from the VPM GPP v20 dataset, which is based on satellite observations.* ***(g)*** *Spatial pattern correlation between the different data sources shown in (a-f).* ***(h)*** *global mean GPP as estimated by the different datasets.*

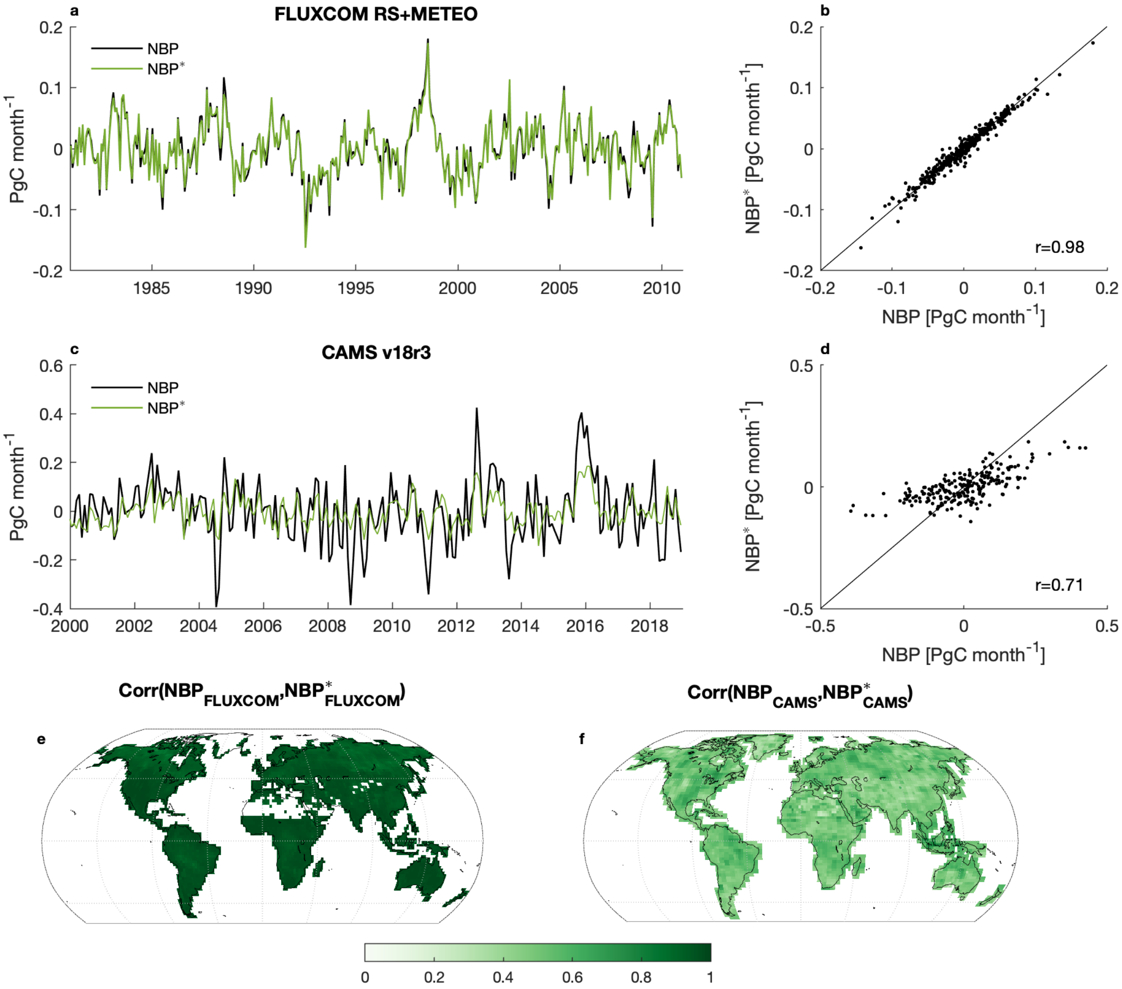

Supplementary Figure 13. Evaluation of the statistical sensitivity analysis (see Methods) when applied to observational datasets. ***a,c)*** *depicts time series of the original global mean NBP and the result of the global mean NBP of the grid-scale month-wise regression (NBP*) (Eq. 1) against the main climatic drivers. The scatter plots* ***(b,d)*** *depict the agreement between these two time series.* ***e-f)*** *show the correlation between NBP and NBP**

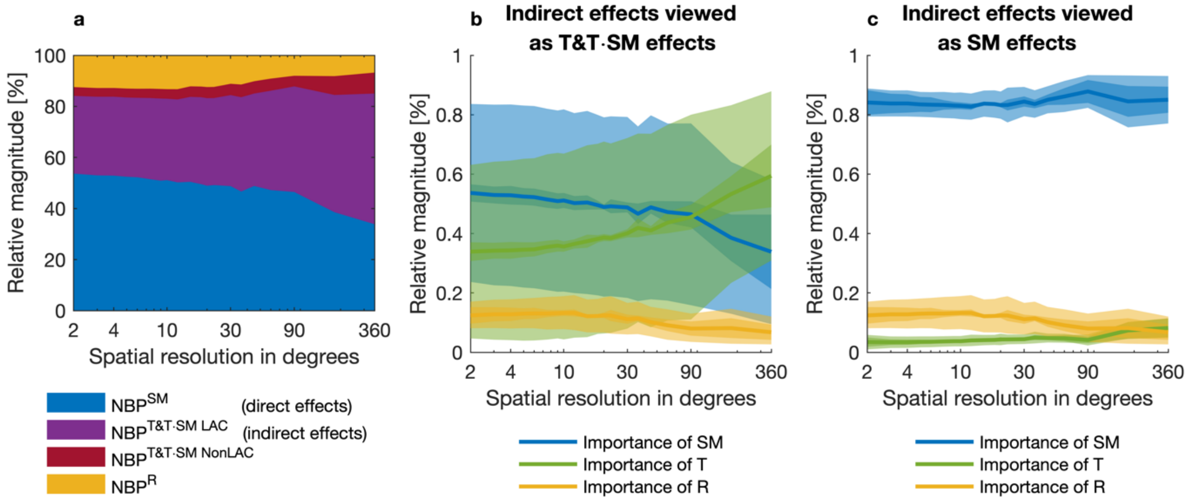

Supplementary Figure 14. Same as Extended Data Fig. 7 but using a different formulation for the sensitivity analysis (NBP = NBP^{SM}+NBP^{T}+ $NBP^{T·SM}$+NBP^{R}, where T·SM represents an interaction term between T and SM).

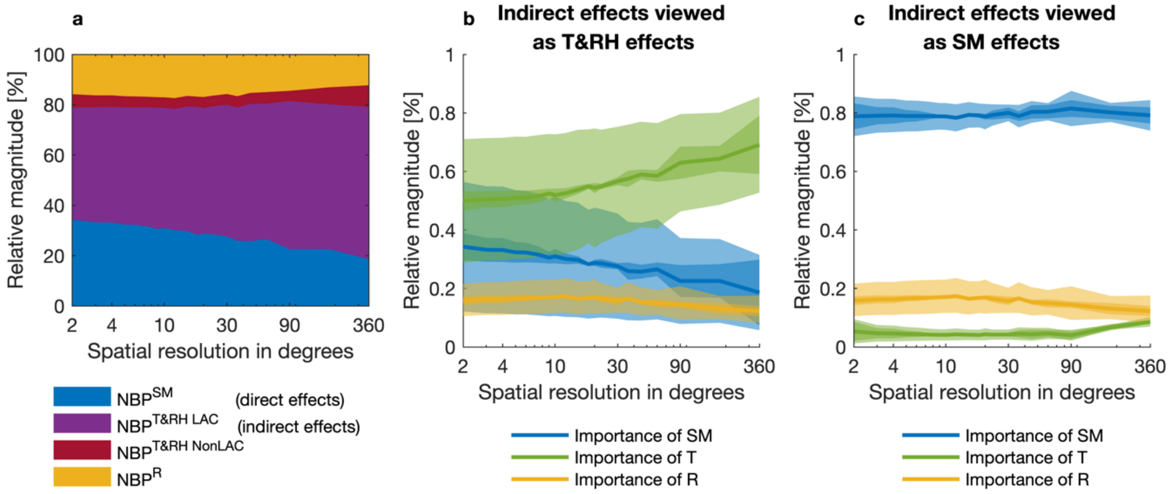

Supplementary Figure 15. Same as Extended Data Fig. 7 but using a different formulation for the sensitivity analysis (NBP = NBP^{SM}+NBP^{T}+ NBP^{RH}+NBP^{R}, where RH indicates relative air humidity).

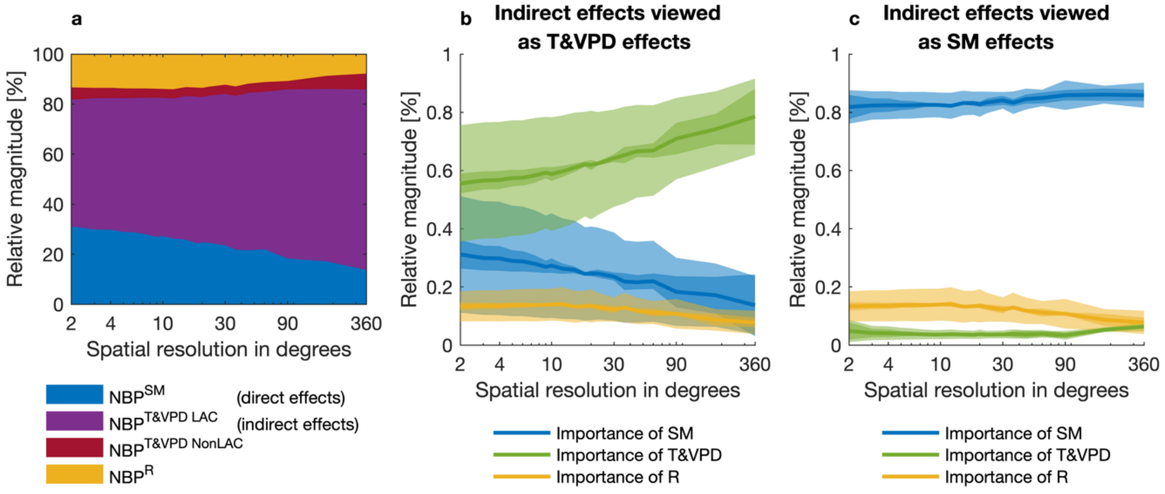

Supplementary Figure 16. Same as Extended Data Fig. 7, but assuming a correlation of zero between $\mathrm{NBP}_{\mathrm{CTL}}^{\mathrm{T\&VPD\ LAC}}$ *and* $\mathrm{NBP}_{\mathrm{CTL}}^{\mathrm{T\&VPD\ NonLAC}}$ *(see Methods) when decomposing* $\mathrm{NBP}_{\mathrm{CTL}}^{\mathrm{T\&VPD}}$.
